# The use of the geometric morphometric method to illustrate shape difference in the skulls of different-aged horses

**DOI:** 10.1007/s11259-020-09779-8

**Published:** 2020-07-23

**Authors:** Tiziana Liuti, Padraic M. Dixon

**Affiliations:** 1 Royal (Dick) School of Veterinary Studies and the Roslin Institute, University of Edinburgh, Midlothian, Scotland EH25 9RG UK; 2 Division of Veterinary Clinical Studies Royal (Dick) School of Veterinary Studies and the Roslin Institute, University of Edinburgh, Midlothian, Scotland EH25 9RG UK

**Keywords:** Geometric morphometric measurements, Computed tomography, Equine skull, Procrustes, Principal component analysis

## Abstract

The geometric morphometrics method (GMM) is a technique to study scale and shape relationships of structures using Cartesian geometric coordinates rather than linear, areal (of area), or volumetric variables. GMM has been of great value in many biological studies, but does not appear to have been used to examine equine skulls.

In this exploratory study, twenty-nine normal equine heads of three different age groups: <5 years old (*N* = 9), 6–15 years old (*N* = 10) and > 16 years old (N = 10) were examined.

Computed tomography (CT) bone window DICOM images were reconstructed into isosurfaces (3-dimensional contoured surfaces), onto which landmarks were added using Stratovan Checkpoint® software. Data from 29 landmarks were analysed using MorphoJ analysis, which applies a Procrustes fit, prior to reducing data dimensionality through principal component (PC) analysis. PCs with and without allometry were considered. Allometric shape described by PC1 accounted for 27% of variance. Loading pertaining to: the pterygoid process, bilaterally; caudal aspect of hard palate; tip of nasal bone; ethmoid sinuses, bilaterally; caudal aspect of the ventral conchal bulla, bilaterally and caudal aspect of the vomer bone suggest that these anatomical structures are predictive of age group. When allometric effects (shape variation explained by size) were removed, PC1 was unable to distinguish horses by age group. Allometric shape differences could distinguish the youngest versus the two older age groups. The potential applications of GMM in equine diagnostic imaging are wide ranging and include the investigation of changes in the equine skull with respect to genetics and characterisation of conformation-related diseases affecting the teeth, jaws and sinonasal compartments.

## Introduction

The study of anatomical shape and its variation is a major topic of medical research. The use of shape analysis is one approach to understanding the causes of variation and the morphological transformation of animals due to evolution, age, disease and selective breeding (Zelditch 2004). Geometric morphometric method (GMM), a technique developed by Bookstein (1984, 1986, 1987, 1991) uses landmarks to explore the morphospace of objects. These landmarks are coordinate points used to represent shape (multiple points required) and are quantified as Cartesian (i.e. x, y, z) coordinates.

GMM was developed to address the shortcomings of simple linear, volume or area-based methods that do not preserve the geometric relationships between the measurements, leading to difficulties in the interpretation of spatial relationships (Bookstein 1991, Glat et al. 1996, Mitteroecker et al. 2004).

One advantage of GMM over traditional morphometric approaches is that it can deconvolute form, separating the effects on shape that are due to, or independent of, size (Bookstein 1984, 1986, 1987). GMM is also more amenable to visual representation of shape variation (Webster and Sheet 2010).

Bookstein applied GMM to studies on craniofacial growth in both humans and animals (Bookstein 1991, Glat et al.^1996^, Mitteroecker et al. 2004) and GMM has since been used in a very wide range of human, animal and plant studies (Komosa et al. 2006, Okumura and Araujo 2014, Muylle et al. 1999).

Equine studies have used traditional morphometric studies to determine neck adiposity in relation to body weight, height, length, girth and abdominal circumference,(Carter et al. 2009) and cerebellar abiotrophy in relation to cerebellar size and cerebellar cerebrospinal fluid space (Cavalleri et al. 2013).

However, GMM does not appear to have been used to study equine skulls. This exploratory study used GMM to assess if the complex bony structures of the equine skull grow homogeneously or at different rates based on age. This three-dimensional morphological information could help our understanding of the pathogenesis of anatomical-related equine head diseases including, overbite, underbite and cheek teeth diastemata that commonly cause severe clinical problems.

The objective of this study was to evaluate the ontogenetic (origination and development) changes that occur in normal equine skulls by using landmarks-based geometric morphometric methods in horses of three different age groups.

Our hypothesis was that there would be ontogenic difference between horses of difference age, with young horses having more skull variability than adult or old horses.

## Material and methods

This study was approved by the University of Edinburgh, Ethical Review Committee.

This exploratory study included the following samples:

### Specimens

Equine heads were obtained from two sources:

### Group a

The heads of 54 horses were freshly obtained from an abattoir. Each head was of unknown history and grossly similar in size to an adult Thoroughbred horse head. Heads were positioned on their mandibles and imaged using a multislice CT scanner (Multislice CT scanner Siemens Volume Zoom, Munich Germany) in a helical scan mode using a 512 × 512 Matrix, 120 Kv, 300 mA, at a slice thickness of 1.5 mm.

Bone windows (H70) were used to review the images at a WW of 4000 Hounsfield Unit (HU) and WL of 1000 (HU). After image acquisition, the heads were frozen (− 20 °C) and then sectioned transversely at 5 cm intervals with a band saw. After thawing, both sides of each section were examined grossly for sinonasal or significant dental abnormalities.

### Group B

Anatomical and CT images were similarly obtained from 36 equine heads with unknown histories that had been freshly obtained from an abattoir by Justin Perkins (Royal Veterinary College, London). CT images were acquired using a 4th generation, Universal Medical System CT scanner, GE light speed ultras, Highland Heights, Ohio, USA, at 1.25 mm slice thickness, 120 kV, 300 mA. The heads and CT images were examined for the presence of sinonasal disease or significant dental abnormalities.

### Eligibility and exclusion criteria for study subjects

Twenty-six of 54 heads in Group A had clinical and/or imaging evidence of dental or sinonasal disease and were thus excluded from the study, leaving 28 heads available in this group. Four of the 36 heads in Group B had clinical and/or imaging evidence of dental or sinonasal disease and were also excluded, leaving 32 heads in this group (a total of 60 normal heads available).

The Computed tomographic images were reviewed by a Dipl.ECVDI (TL) and the clinical examination of each head by a Dipl.ECVD (equine) (PMD).

Therefore data selection and recording was made by the combination of both imaging and clinical evidence (TL and PMD). The analysis including statistical test was mainly conducted by the first author (TL) using software designed for GMM analysis (Stratovan CheckPoint®; 3 WIN ×64)^a^ and MorphoJ^b^ (Klingenberg 2011).

### Comparison of head dimensions with those of horses of known breed

In order to compare the head sizes in the current study population with head sizes of known breeds, CT images of 12 Thoroughbreds of known age (0–5 years old [*N* = 4]; 6–15 years old [N = 4]; >16 years old [N = 4]) that had undergone CT head imaging for clinical reasons other than sinonasal disorders were measured to obtain their head dimensions.

### Head linear dimensions and volumes

Head “length” (i.e. distance from the caudal aspect of the orbit to the nasoincisive notch) was measured from CT sagittal reconstructions; head “width” (width of the hard palate at the level of Triadan 06) was measured from CT dorsal reconstructions and head “height” (distance from the hard palate to the dorsal aspect of the maxillary bone at the level of the orbit) was measured using CT sagittal reconstructions. These three measurements were multiplied together to produce a measurement of head “volume” (Liuti et al. 2016).

### Statistical analyses of head sizes

A paired T-test (Minitab®)^c^was used to assess any differences in dimensions between the cadaver heads used in this study and the 12 control Thoroughbred horses.

The 60 heads were aged by clinical incisor examination using standard guidelines along with CT measurements of cheek teeth reserve crown and root length. Heads were placed into three age groups: <5 years old (Group 1: *N* = 15), 6–15 years old (Group 2: *N* = 21) and > 16 years old (Group 3: *N* = 24).

### Image-based morphometrics

Bone window CT data of the 60 heads were transferred as DICOM images to Osirix® (www.osirix-viewer.com) imaging software which was used to perform multiplanar image reconstructions and to evaluate the image quality. Isosurfaces of each skull were generated from their constituent DICOM images using Stratovan CheckPoint® software which allows collection of x, y and z coordinates from landmarks placed on skull images. Twenty-nine heads were selected for this study: 0–5 years old (Group 1: *N* = 9), 6–15 years old (Group 2: *N* = 10) and > 16 years old (Group 3: N = 10) based on the accurate identification of all anatomical landmarks (see below) after the isosurface was generated from the DICOM images.

**Fig. 1:**
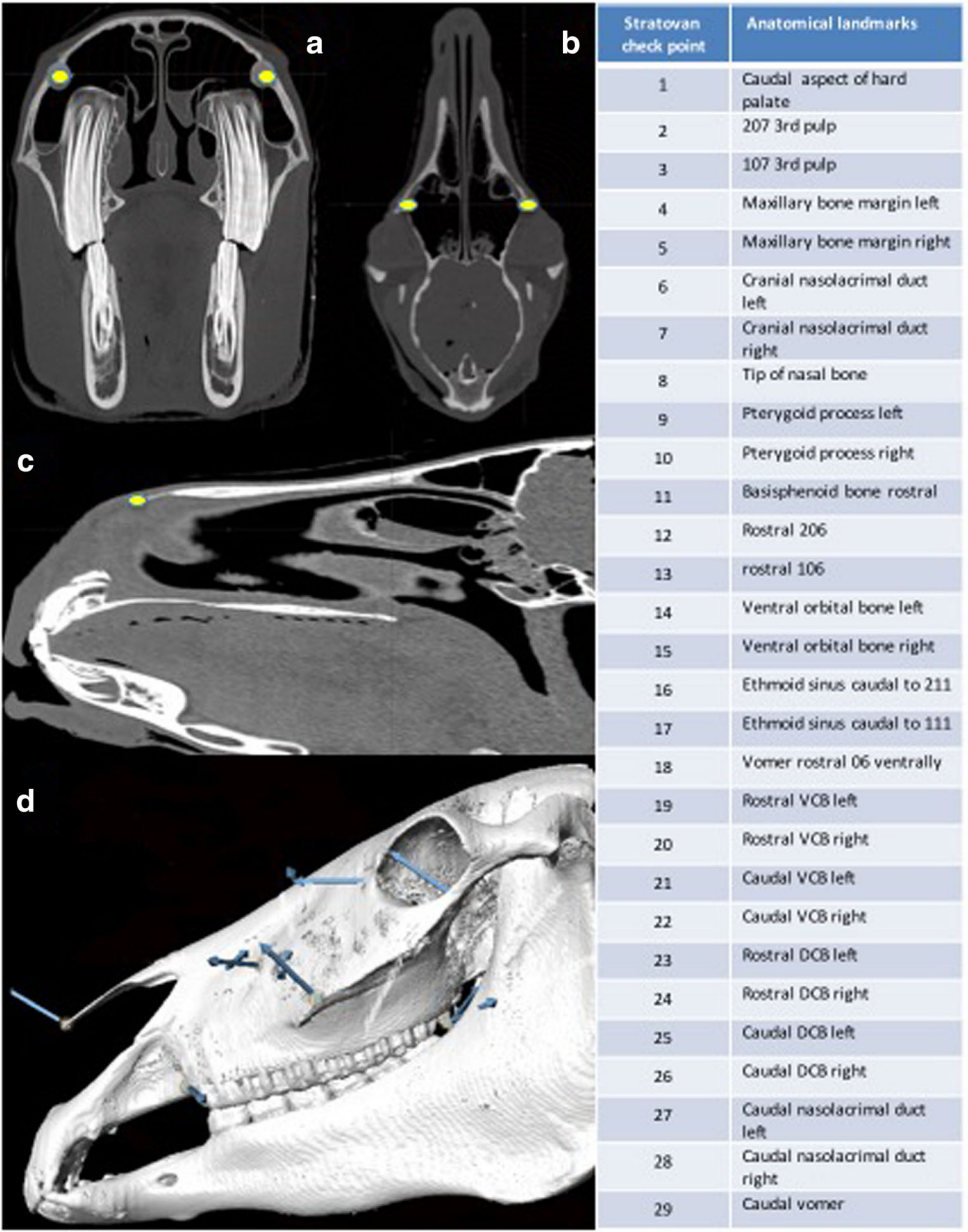
Anatomical landmarks. A) Stratovan checkpoint transverse slice reconstruction: note landmarks (yellow dots) at the level of the nasolacrimal duct ostia, left and right. B) Stratovan checkpoint dorsal slice reconstruction: note landmarks (yellow dots) at the level of the maxillary bone lateral margins, left and right. C) Stratovan checkpoint sagittal slice reconstruction (note landmark (yellow dot) at the level of the tip of nasal bone. D) Stratovan checkpoint lateral isosurface showing external landmarks VCB: Ventral Conchal Bulla, DCB: Dorsal Conchal Bulla.

Twenty-nine homologous landmarks (this number coincidental to the number of skulls) were used in each skull (Fig. 1 A-D). Each landmark was uniquely identified using anatomical nomenclatures to denote those on the external and internal skull surfaces (the latter including: the ventral and dorsal conchal bullae, individual tooth pulps, nasolacrimal duct ostium, and caudal aspect of the hard palate), to establish if the morphology of certain skull sites showed age-related variability.

Placement of landmarks was performed on sagittal, dorsal or transverse slice windows or on the isosurface.

### Geometric Morphometrics

Cartesian landmark coordinates were reformatted using custom R scripts and loaded into MorphoJ (Klingenberg 2011) for processing. Briefly, a Procrustes fit was used to superimpose, translate, rotate, and uniformly rescale coordinates. The degree of rescaling required to minimise the sum of the squared distances between landmarks (termed centroid size) was used as a proxy for animal scale in this study. A covariance matrix was generated either from coordinates or regression residuals (see the next section). From these matrices, PC analysis was used to describe correlated landmark covariances into orthogonal, uncorrelated systems (“PCs”, also termed eigenvectors). In effect, PCs summarise the total variation described across the dataset, with each successive component describing smaller tranches of uncorrelated variance than the preceding PC (i.e. PC1 > PC2, PC2 > PC3…).

To remove allometric effects, a multivariate regression of Procrustes coordinates against the log-transformed centroid size was used. The resulting residuals produced by the regression were used to generate a covariance matrix, which was analyzed by PC as described above. The multivariate regression was tested with permutation tests against the null hypothesis.

Due to the relatively small contributions of PCs greater than PC2, only the first and second PCs (PC1 and PC2, respectively) analyses were considered in this study.

In order to correct for the effect of size on shape, a regression analysis on log- transformed centroid size with a permutation test was performed. This analysis uses the null hypothesis of complete independence between the dependent and independent variables^16^; in this study between the landmarks for each age group and the log-centroid size. A total of 10,000 random permutations were conducted and the multivariate regression result was highly significant (*p* value: <0.0001).

A Discriminant Function Analysis (DFA) was also considered in the data evaluation to determine any variable present between the three different groups: Group 1 vs Group 2, Group 1 vs Group 3 and Group 2 vs Group 3.

## Results

No significant difference was found between head length (*p* = 0.196), width (*p* = 0.942), height (*p* = 0.086) or volumes (*p* = 0.829) between the cadaver heads used in this study and the 12 control Thoroughbred horses (*p* < 0.05).

### Principal components analysis (PCA)

#### a) PCA with allometry

**Fig. 2:**
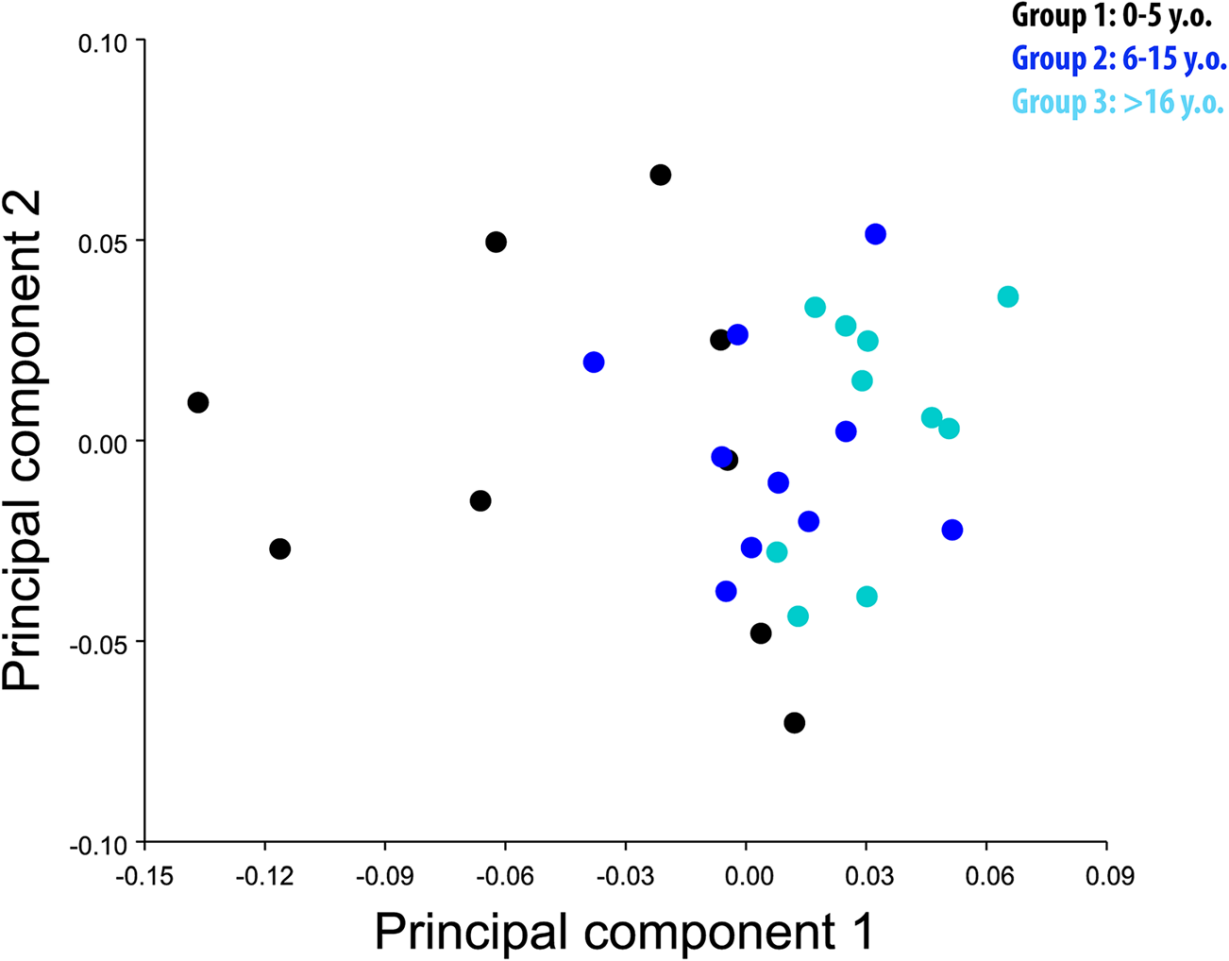
PCA 1 vs PCA 2 with allometry. There is obvious overlap between Groups 2 (light blue) and 3 (blue) but much distinction between Groups 1 (black) and 3 (blue).

**Fig. 3:**
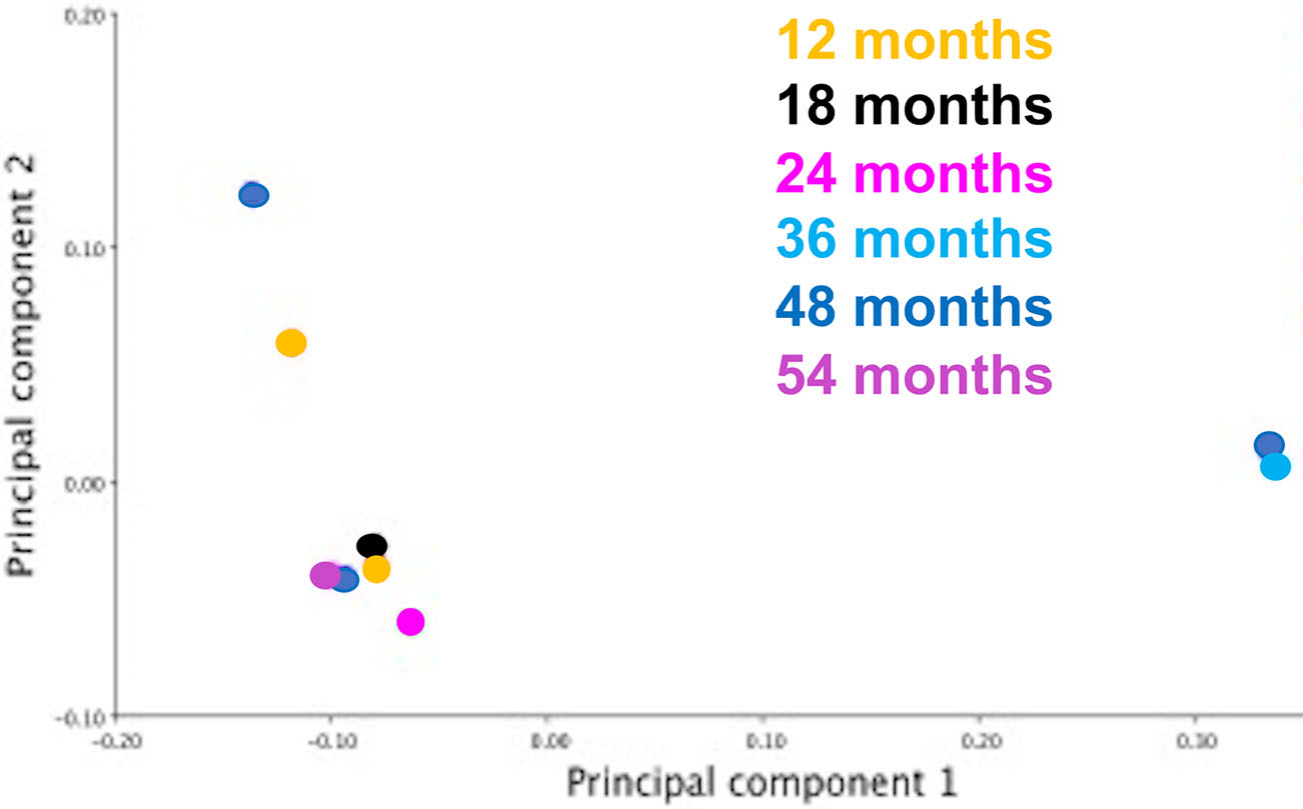
PC1 vs PC2 within Group 1 divided by age in month (colour coded). Two individuals (on right of figure) are located distant from the rest of the group, most likely due to their different skull morphology. This figure shows the relatively large morphospace occupied by these young horses

PC1 and PC2 findings showed clustering of individuals based on group membership (i.e. age), demonstrating minimal overlap between Groups 1 and 2 and no overlap between Groups 1 and 3. However, the overlap between Groups 2 and 3 was substantial. These results could be interpreted as showing a high variability in skull shape in young horses (Group 1; <5 y.o.), while the skull form of adult (Group 2; 6–15 y.o.) and old horses (Group 3; >16 y.o.) is comparatively fixed. A further explanation for the difference between age groups is provided by the large morphospace the young group occupies in PC1, in relation to Groups 2 and 3 (Fig. 2). A further PC1 vs PC2 run within Group 1 by their age in months to assess for any trend in morphospace (Fig. 3) showed two horses (36 and 48 months old) located further away from the other 7, most likely due to different skull morphology.

**Fig. 4:**
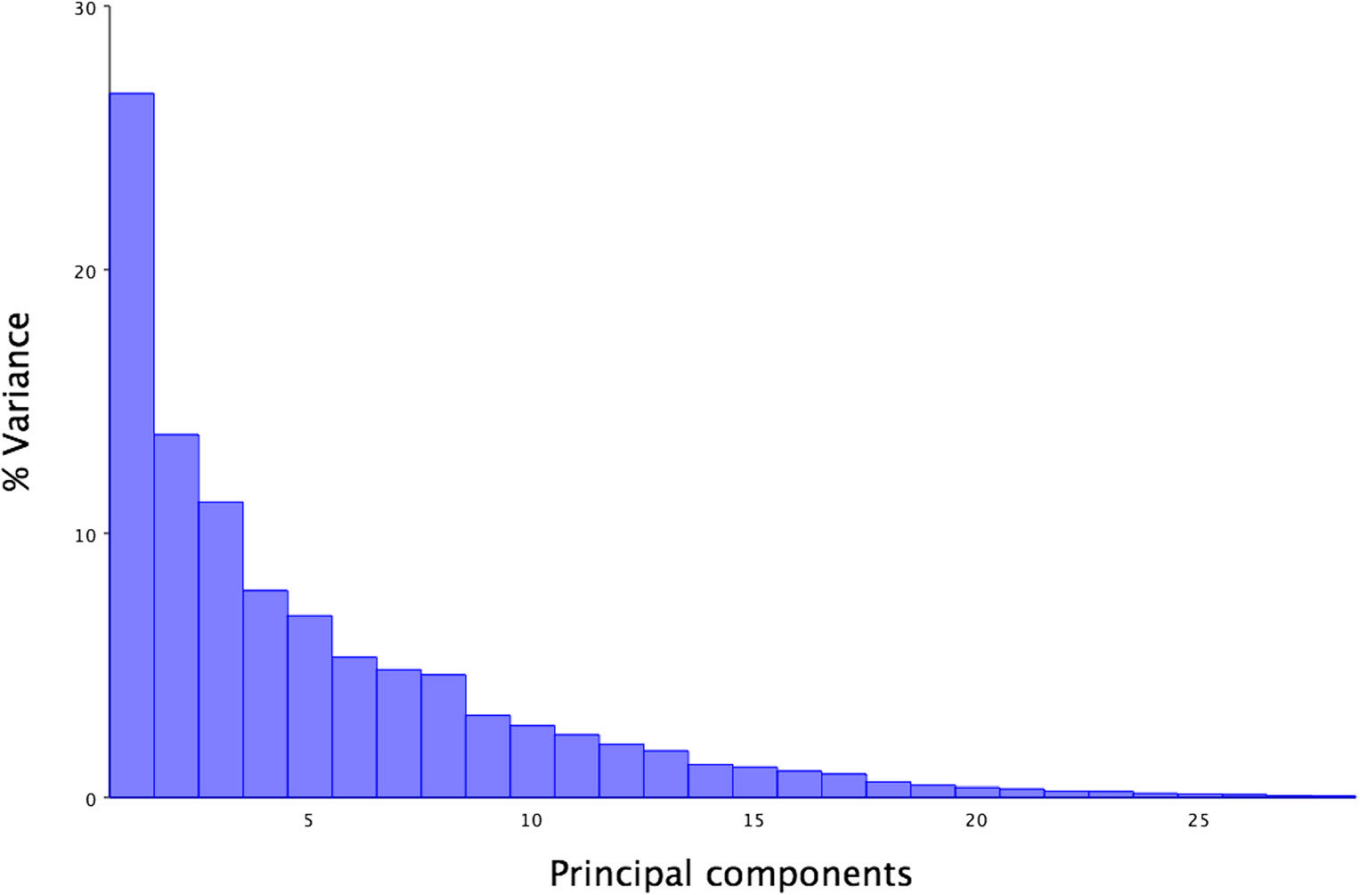
Eigenvalue graph of PCA1 with allometry; note the 27% variance in PC1, with much lower variance in the other Principal Components

**Fig. 5:**
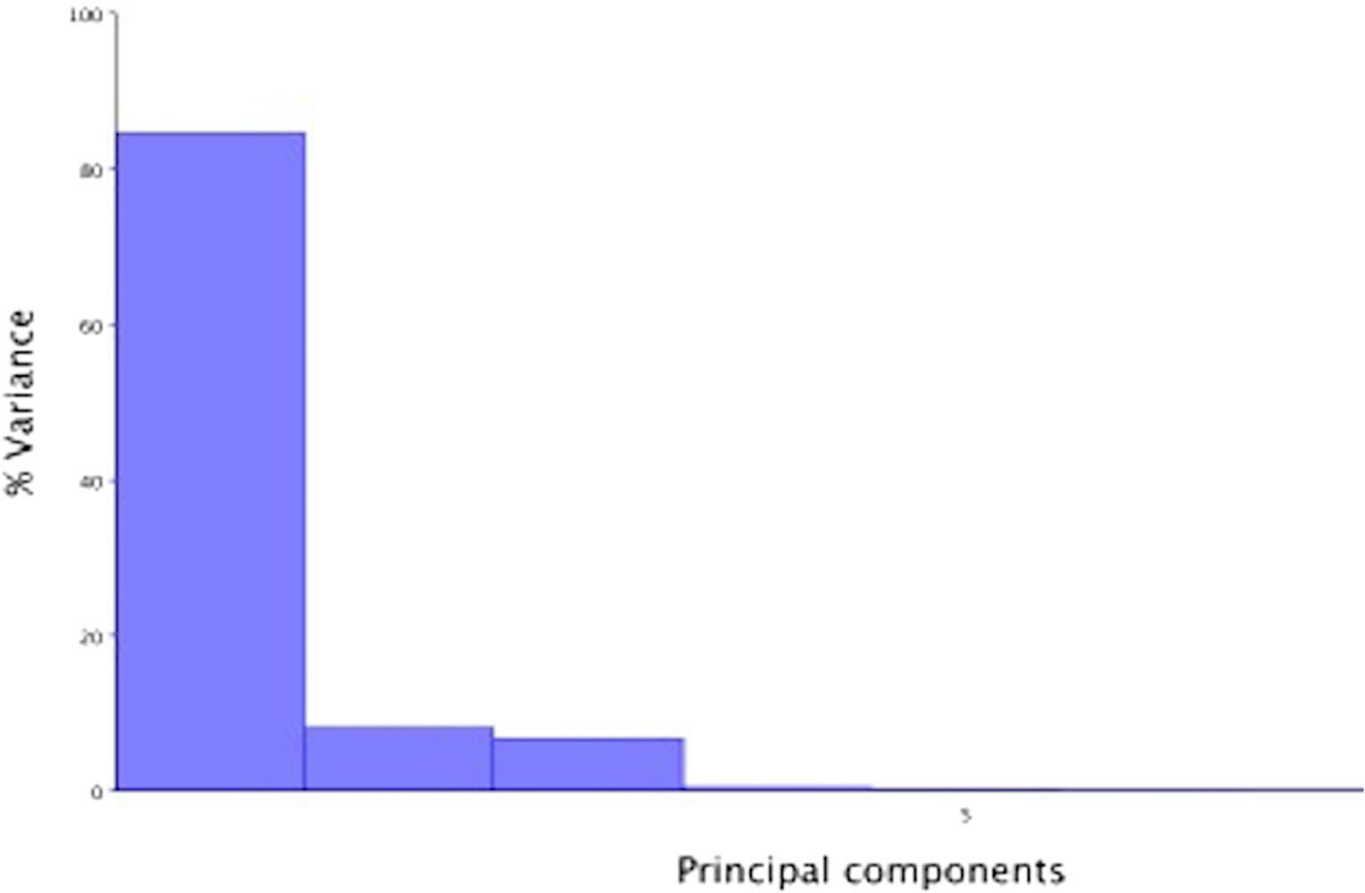
Eigenvalue graph of PCA1 with allometry within Group 1: note the 85% variance in PC1 with minimal (<8%) variance in PC2, PC3 and PC4

In the eigenvalues graph, PC1 allometry explained 27% of the shape variation between all 29 skulls (Fig. 4) but a further eigenvalue graph created within Group 1, showed that 85% of their skull shape variation could be explained by PC1 allometry (Fig. 5).

**Fig. 6:**
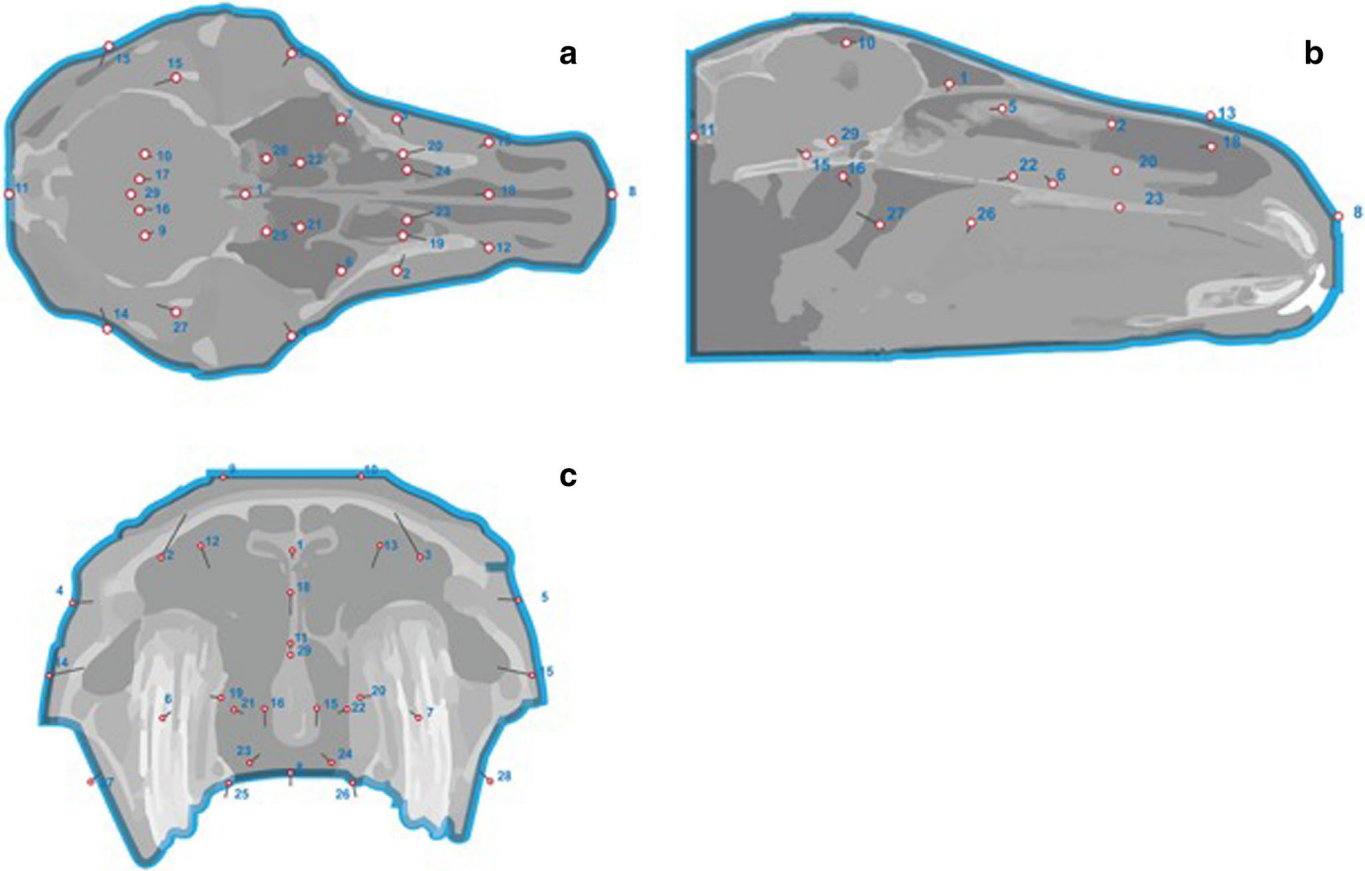
Graphical representation of PCA 1 with allometry in all 29 horses in dorsal, sagittal and transverse reconstructions. Landmarks are represented by “lollipops” with their length and direction indicative of the degree and direction of anatomical variation

A graphical representation of PCA shows variability of the chosen landmarks in different planes (dorsal, sagittal and transverse, respectively) (Fig. 6 (A, B, C). In particular, the following landmarks: caudal aspect of hard palate, pterygoid process (left and right), basisphenoid bone, and rostral and caudal aspects of vomer showed minimal changes in direction or size.

#### b) PCA without allometry

**Fig. 7:**
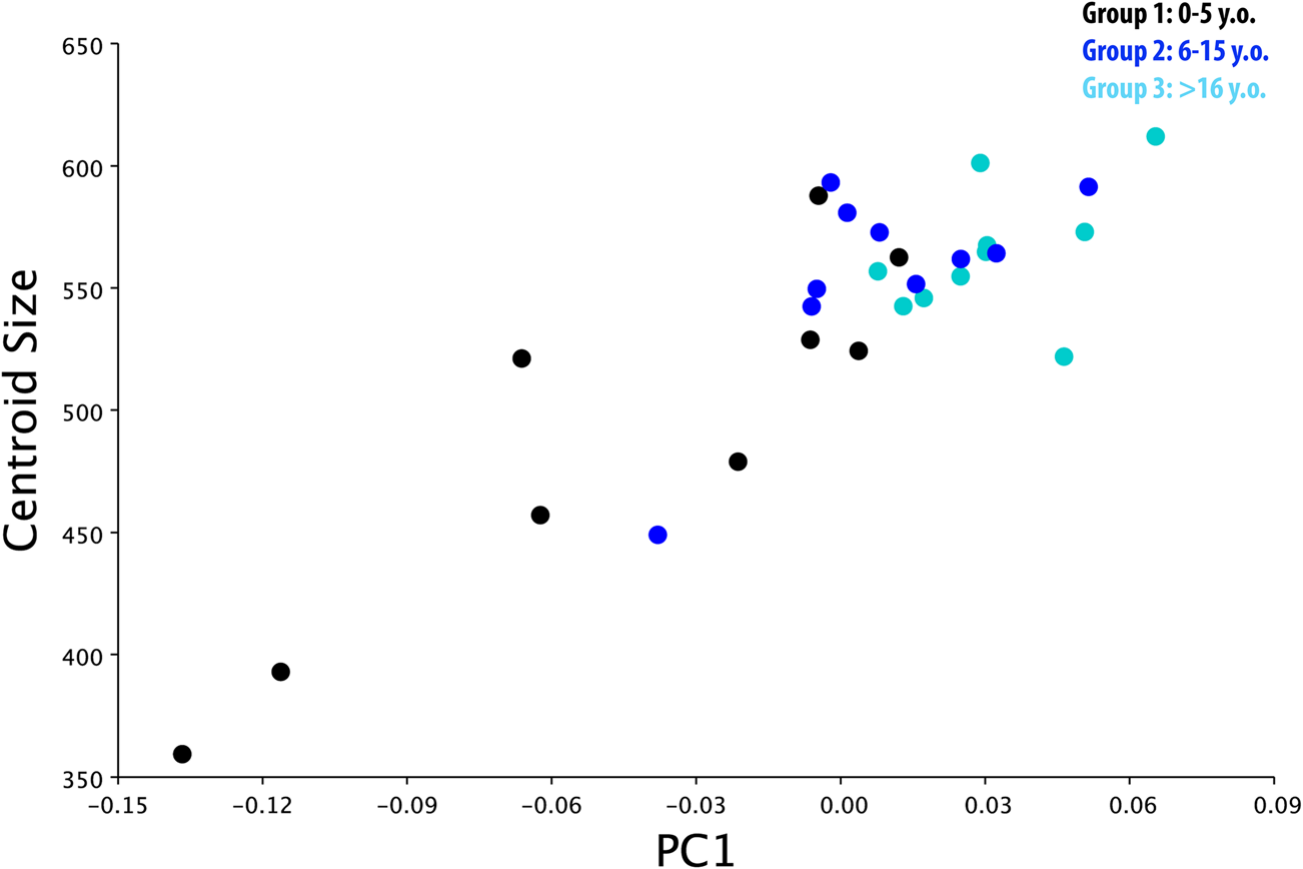
Graphical representation of PC1 without allometry in all 29 horses. The morphospace occupied by each age group overlaps. Groups 2 (light blue), Group 3 (blue) and Groups 1 (black)

PCA without allometry showed no obvious clustering of individuals based on group membership, but showed clustering between Groups 1 and 2, Groups 2 and 3, and Groups 1 and 3, indicating poor separation between the different age groups (Fig. 7).

### Discriminant functional analysis

Discriminant Functional Analysis shows direction and associated point variability between age groups (Group 1 vs 2; Group 1 vs Group 3; Group 2 vs 3) in different anatomical orientations (dorsoventral, sagittal and transverse planes).

Two landmarks (pterygoid process; left and right) showed no variability between Groups 1 and 3, whilst three landmarks (caudal aspect of hard palate; pterygoid process, left and right) showed no variability between Groups 1 and 2. Eight landmarks (tip of nasal bone; pterygoid process (left and right) ethmoidal sinus caudal to 211 and 111; caudal aspect of VCB (left and right) and caudal aspect of vomer bone showed no variability between Groups 2 and 3. These results highlight the variability of the landmarks between age groups, with horses in Group 1 showing most variability in landmarks and only 2/29 landmarks not showing any variability.

## Discussion

This appears to be the first publication to utilise GMM to investigate ontogenic shape differences in normal equine skulls in horses of different age and showed shape changes that were related to skull growth. These changes within the growing equine skull were not uniform with the allometric shape of skulls of horses under 5 years old differing substantially from older age groups.

In the current study, PCA graphic representation showed a larger morphospace occupied by young horses (Group 1) in comparison to the older horses (Groups 2 and 3) (Fig. 2), whilst PCA within Group 1 showed seven individuals with similar and two individuals with very different morphological characteristics (Fig. 3). A possible explanation for the major morphological differences of those two young horses could be related to their breeds, because some (“cold-blooded”) ponies have heads similar in size to Thoroughbreds. Further studies on groups of young horses of known breeds would better define interbreed differences in head morphometrics.

On PCA graphic representation, Groups 2 and 3 occupied a smaller morphospace than Group 1, most likely due to lesser skull shape variability between these two older groups of horses.

The second part of the study investigated possible association between size and shape of the skulls. A PCA graphical representation without allometry was performed using multivariate regression analysis with an associated permutation test. This showed poor differentiation between different age groups, in particular between Groups 1 and 3 where minimal overlap was visible. In this graph, a large morphospace is occupied by Group 1 in relation to Groups 2 and 3. However, in order to test the statistical significance of the association between size and shape, a permutation test was carried out. This permutation test implemented the null hypothesis of independence between size and shape (Good 2000) without making assumptions about the data distribution and showed a statistically significant positive correlation between size and shape, thus making allometry essential in differentiating individuals from the different age group.

In the third part of the analysis, a DFA was calculated to examine differences and relationships between age groups. DFA has been used in multiple human and animal.

studies, including an investigation of the relationship between cranial morphology and body build in horses (Komosa et al. 2006).

Much variability was found in landmark orientation in Group 1 (<5y.o.) vs Groups 2 and 3 (>5 y.o.) horses, with lesser variability found between Groups 1 and 2, and Groups 2 and 3. In particular, the results revealed that PC loadings between Groups 1 and 3 were located at the pterygoid process bilaterally, while PC loadings between Groups 1 and 2 were located at the pterygoid process bilaterally and also at the caudal aspect of the hard palate. PC loadings between Groups 2 and 3 were located at multiple locations (tip of nasal bone, pterygoid process, bilaterally; ethmoid sinus, bilaterally; caudal ventral conchal bulla, bilaterally; and the caudal aspect of the vomer).

These findings showed that the variation in equine skull shape between age groups is not uniform, but more pronounced in specific anatomical areas. However, the pterygoid bone showed minimal variation and appeared to be at a constant location within the three groups. Vidic (1968) examined the pterygoid process in humans and animals (including dogs, sheep, pigs, cattle and horses) and described the length of the pterygoid process as a function of the variation of the sphenoid angles and also showed slight age-related variation in the human pterygoid process length (Vidic 1968).

Future GMM studies could investigate changes in equine skulls of defined known age, breed and gender in relation to their genotype to characterize breed-related disorders affecting teeth and sinonasal compartments. For example, miniature horses are predisposed to dental overcrowding due to their small skull and large teeth conformation, predisposing them to severe dental and sino-nasal problems.

Overjet/overbite (“Parrot mouth”), a rostral malocclusion of the upper incisors in relation to the lower incisors is the most common equine congenital craniofacial abnormality (Easley et al. 2016, De Bowes and Gaughan 1998). Overjet/overbite is an inherited disorder and Thoroughbred horses are more predisposed than other breeds (Dixon et al. 2014); as of yet, it remains unclear if this condition is due to mandibular brachygnathism or maxillary prognathism. DNA and associated GMM analysis of an affected population could also identify the relationship between the genotype and phenotype responsible for this condition.

Cheek teeth diastemata, the most common, painful equine dental disease affecting 50% of UK horses (Walker et al. 2012), is believed to be due to an imbalance between jaw size and cheek tooth size and orientation. This disorder could also be effectively examined by GMM in order to better determine the morphological characteristics of this disease.

Previous GMM studies have examined the diversity of populations of late glacial horses in Western Europe by detailed morphological analysis of their metacarpal and metatarsal bones (Bignon et al. 2005). Broader GMM studies using multiple body measurements of the size and shape of morpho- functional traits in Spanish Arab horses showed no shape differences in horses bred for different purposes but a significant difference in size between morphological and endurance aptitude (Cervantes et al. 2009). A further equine GMM study showed significant interbreed, but no age-related differences on occlusal enamel folding of equine Triadan 06 and 11 teeth (Seetah et al. 2014).

The main limitation of the study was the lack of histories or signalment information available for any of these horses, whose skulls were similar in size to Thoroughbred skulls.

Gross pathological and CT examinations did not show any evidence of bony malformation secondary to localised or generalised disease, such as nutritional secondary hyperparathyroidism (Joyce et al. 1971).

## Conclusion

This GMM study showed shape changes related to equine skull growth. These changes within the growing equine skull are not uniform with the allometric shape of skulls of horses under 5 years old differing substantially from older age groups.

**Footnotes.**

^a^ Stratovan CheckPoint®; 2017.04.21.1249 3 WIN ×64.

^b^ MorphoJ: Klingenberg C.P. MorphoJ: an integrated software package for geometric morphometrics. *Molecular ecology Resources* 2011; 11: 353–357.

^c^ Minitab®: Minitab 17 Statistical Software (2010).

### List of authors contributions

Conceptualization: Tiziana Liuti.

Methodology: Tiziana Liuti, P.M Dixon.

Formal Analysis and Investigation: Tiziana Liuti.

Writing-original draft preparation: Tiziana Liuti, P.M Dixon.

Writing-review and editing: Tiziana Liuti, P.M Dixon.

Funding Acquisition; N/A.

Resources: N/A.

Supervision: P.M Dixon.

## List of abbreviations

**DFA**: Discriminant Function Analysis
**GMM**: Geometric Morphometric Method
**PC**: Principal Component
**PCA**: Principal Component Analysis

## References

[CR1] Bignon O, Baylac M, Vigne J.D, Eisenmann V. (2005) Geometric morphometric and the population diversity of late glacial horses in Western Europe (Equus caballus arcelini): phylogeographic and anthropological implication. J Archeol Sci 32: 375–391

[CR2] Bookstein FL (1984). A statistical method for biological shape comparisons. J Theor Biol.

[CR3] Bookstein FL (1986). Size and shape spaces for landmark data in two dimensions. Stat Sci.

[CR4] Bookstein FL (1987). Describing a craniofacial anomaly: finite elements and the biometrics of landmark locations. Am J Phys Anthropol.

[CR5] Bookstein FL (1991). Introduction. Morphometric tools for landmark data.

[CR6] Carter R.A, Raymond J.G, Burton Staniar W, Cubitt T.A, Harris P.A.(2009) Apparent adiposity assessed by standardised scoring system and morphometric measurements in horses and ponies. Veterinary Journal 179: 204–210 10.1016/j.tvjl.2008.02.029 18440844

[CR7] Cavalleri JM, Metzger J, Hellige M, Lampe V, Stuckenschneider K, Tipold A, Beineke A, Becker K, Disti O, Feige K (2013). Morphometric magnetic resonance imaging and genetic testing in cerebellar abiotrophy in Arabian horses. BMC Vet Res.

[CR8] Cervantes I, Baumung R, Molina A, Druml T, Gutierrez J.P, Solkner J, Valera M. (2009) Size and shape analysis of morphofunctional traits in the Spanish Arab horse. Livest Sci 125: 43–49

[CR9] De Bowes RM, Gaughan EM (1998). Congenital dental disease of horses. Veterinary Clinic North America Equine Practice.

[CR10] Dixon PM, Ceen S, Barnett T, O'Leary JM, Parkin TD, Barakzai S (2014). A long-term study on the clinical effects of mechanical widening of cheek teeth diastemata for treatment of periodontitis in 202 horses (2008–2011). Equine Veterinary Journal.

[CR11] Easley J, Dixon PM, Reardon RJM (2016). Orthodontic correction of overjet/overbite (‘parrot mouth’) in 73 foals (1999-2013). Equine Vet J.

[CR12] Glat PM, Freund RM, Spencer JA, Levine J, Noz M, Bookstein FL, Mc Carthy JG, Cutting CB (1996). A classification of plagiocephaly utilizing a three-dimensional computer analysis of cranial base landmarks. Annual of Plastic Surgery.

[CR13] Good P. (2000) Permutation tests: a practical guide to resempling methods for testing hypothesis 2nd edn springer New York 1-12p

[CR14] Joyce JR, Pierce KR, Romane WM, Baker JM (1971). Clinical study of nutritional secondary hyperparathyroidism in horses. Journal of American Veterinary Medicine Association.

[CR15] Klingenberg CP (2011). MorphoJ: an integrated software package for geometric morphometrics. Mol Ecol Resour.

[CR16] Komosa M, Molinski K, Godynicki S (2006). The variability of cranial morphology in modern horses. Zool Sci.

[CR17] Liuti T, Reardon R, Smith S, Dixon P.M. (2016) An anatomical study of the dorsal and ventral nasal Conchal bullae in normal horses: computed tomographic anatomical and morphometric findings. Equine Vet J 48: 749–755 10.1111/evj.12516 26440763

[CR18] Mitteroecker P, Guz P, Bernhard M, Schaefer K, Bookstein FL (2004). Comparison of cranial ontogenic trajectories among great apes and humans. J Hum Evol.

[CR19] Muylle S, Simoens P, Lauwers H (1999). Age-related morphometry of equine incisors. *Zentralblatt Fur Veterinarmedizin*. Reihe A.

[CR20] Okumura M, Araujo A.G.M. (2014) Long-term cultural stability in hunter– gatherers: a case study using traditional and geometric morphometric analysis of lithic stemmed bifacial points from southern Brazil. J Archeol Sci 45: 59–71

[CR21] Seetah K, Cucchi T, Dobney K, Barker G (2014). A geometric morphometric re-evaluation of the use of dental form to explore differences in horse (Equus caballus) population and its potential zooarchaeological application. J Archaeol Sci.

[CR22] Vidic B (1968). The variation in length of the pterygoid process as a function of the variations of the sphenoid angle. Anat Rec.

[CR23] Walker H, Chinn E, Holmes S, Barwise-Munroe L, Robertson V, Mould R, Bradley S, Shaw D.J. and Dixon P.M. (2012) Study of the prevalence and some clinical characteristics of equine cheek teeth diastemata in 471 horses examined in a UK first opinion equine practice (2008–2009). Vet Rec 171: 44 10.1136/vr.100829 22706040

[CR24] Webster M, Sheet H.D. (2010) A practical introduction to landmark-based geometric morphometrics. The paleontological Society Papers 16: 163–188

[CR25] Zelditch ML (2004) Introduction: in geometric morphometric for biologist. Elsevier Ltd:1–8p

